# Possibilities and efficiency of MSC co-transfection for gene therapy

**DOI:** 10.1186/s13287-024-03757-6

**Published:** 2024-05-23

**Authors:** Sina Christoffers, Lisa Seiler, Elena Wiebe, Cornelia Blume

**Affiliations:** 1 Institute for Technical Chemistry, Leibniz University Hannover, Callinstr. 3-5, 30167 Hannover, Germany; 2 Cluster of Excellence Hearing4all, Hannover, Germany

**Keywords:** Mesenchymal stem cells, Genetic modification, Viral transfection, Lipofection, Electroporation, CRISPR/Cas9

## Abstract

Mesenchymal stem/stromal cells (MSCs) are not only capable of self-renewal, trans-differentiation, homing to damaged tissue sites and immunomodulation by secretion of trophic factors but are also easy to isolate and expand. Because of these characteristics, they are used in numerous clinical trials for cell therapy including immune and neurological disorders, diabetes, bone and cartilage diseases and myocardial infarction. However, not all trials have successful outcomes, due to unfavourable microenvironmental factors and the heterogenous nature of MSCs. Therefore, genetic manipulation of MSCs can increase their prospect. Currently, most studies focus on single transfection with one gene. Even though the introduction of more than one gene increases the complexity, it also increases the effectivity as different mechanism are triggered, leading to a synergistic effect. In this review we focus on the methodology and efficiency of co-transfection, as well as the opportunities and pitfalls of these genetically engineered cells for therapy.

**Graphical abstract:**

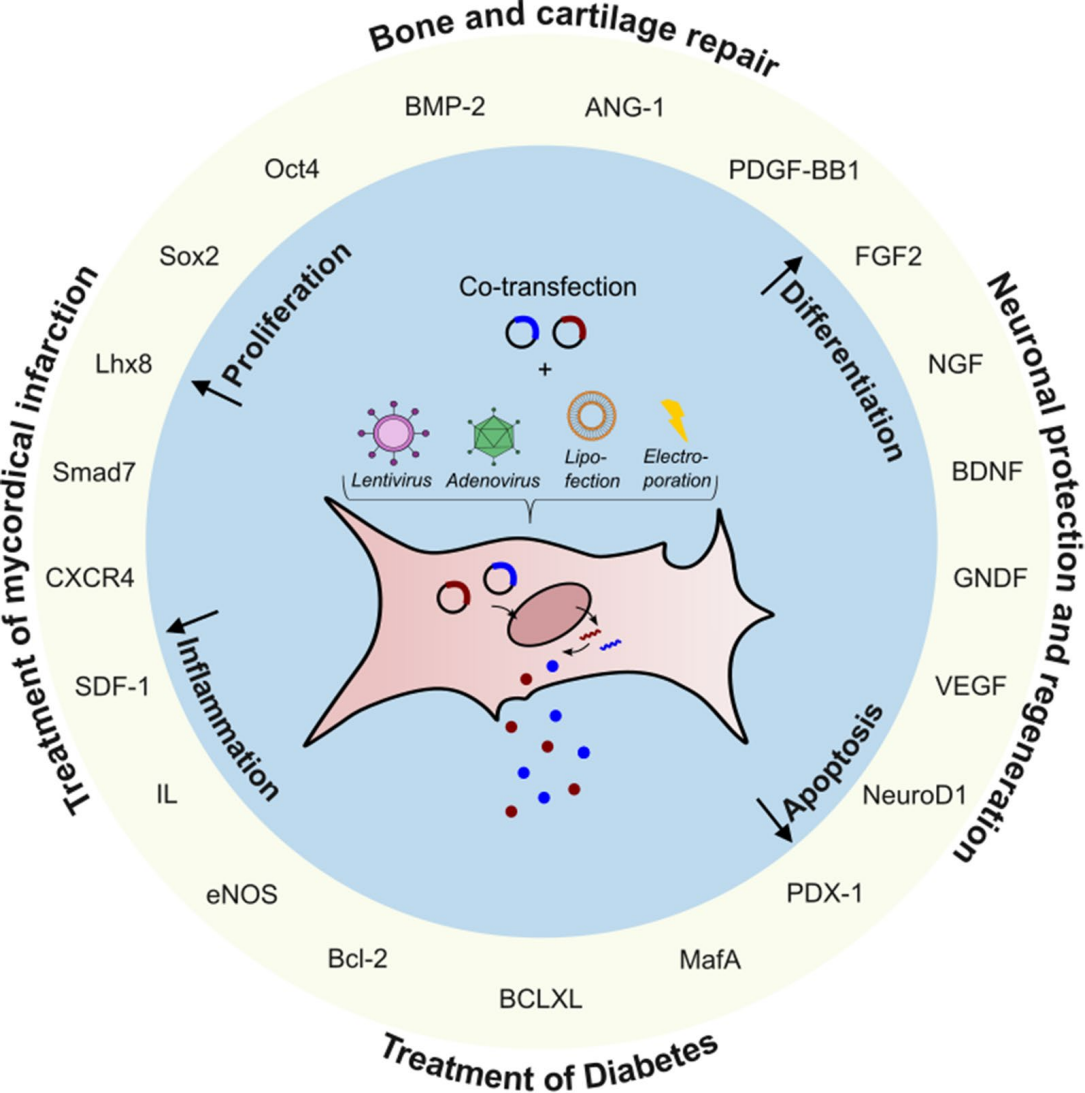

## Motivation

Co-transfection involves the introduction of multiple nucleic acids into the cell. Genetic manipulation of mesenchymal stem/stromal cells (MSCs) with more than one gene could be useful to multi-factor differentiate cells for tissue engineering or to make target genes sensitive to specific regulatory systems. Furthermore, genome editing by CRISPR/Cas9 is also fundamentally based on the parallel introduction of the nuclease Cas9 and a specific guide RNA.

MSCs are promising candidates for gene therapies, as they have immunomodulatory properties, colonize injured tissue sites, are less immunogenic and tumorigenic than induced pluripotent stem cells, and are relatively easy to isolate, expand and differentiate.

It is an urgent problem, that especially MSCs are not as susceptible to co-transfection as cell lines or primary cell types of lower complexity. Nevertheless, for clinical therapies, the low immunogenic and relatively undifferentiated phenotype of MSCs is crucial in terms of immunogenic tolerance in the patients treated with MSC therapies. The review is therefore dedicated to giving comprehensive information on all issues of co-transfection and choice of MSC subtypes, to picture a feasible concept of MSC co-transfection possibilities.

Therefore, in this review, we will focus on the use of MSCs in co-transfection procedures. First, we introduce MSCs, and explain their origin and potency for therapy, before we discuss the challenge of MSC heterogeneity and donor variability with its impact on transfection efficiency. Second, we, we provide an overview of the transfection methods and discuss the resulting transfection efficiencies. Finally, we show the applications frequently found in the literature regarding co-transfection and discuss the implications of using genetically engineered MSCs for gene therapy.

## MSCs-origin and cell sources

MSCs are multipotent adult stromal cells that originally form the mesenchyme, a part of the embryonic connective tissue. However, they are found in almost all postnatal tissue types. Isolated cells consist of mixed populations of progenitor cells, multipotent stem cells and stem cells with varying degrees of differentiating capacity and differentiated cells [1]. According to the International Society for Cellular Therapy ISTC, MSCs must fulfil minimum criteria: (i) MSCs must be able to adhere to plastic, (ii) they must express the surface markers CD105, CD73 and CD90 but not CD45, CD34, CD14, CD19 and HLA-DR, (iii) MSCs must differentiate in vitro into adipocytes, chondrocytes and osteocytes [2].

The term mesenchymal stem cell, which was introduced by Caplan in 1991, is still used widely in the literature, although these cells fail to regenerate tissues in vivo and only a small subset of isolated cells are bona fide stem cells. Because of this the ISTC termed the cells under multipotent mesenchymal stroma cells. However, to reflect the function of MSCs, Caplan introduced another term for MSCs as medicinal signal cells [3]. As the term is not commonly used, we think the combined definition of mesenchymal stem/stromal cells to be the most accurate.

Currently, 1448 clinical trials with MSCs are registered [4], however, some studies have divergent outcomes. Transplanted MSCs face unfavorable microenvironmental factors, especially in ischemic tissue. Additionally, some patients do not respond to MSC-based therapy. This could be due to the fact, that MSCs are very heterogeneous, mostly depending on their tissue origin and environment [5] as well as on the donor’s age [6], gender and health status [7]. MSCs exhibit a high plasticity, and culture and experimental conditions as well as cryopreservation can alter the phenotype.

**Fig. 1:**
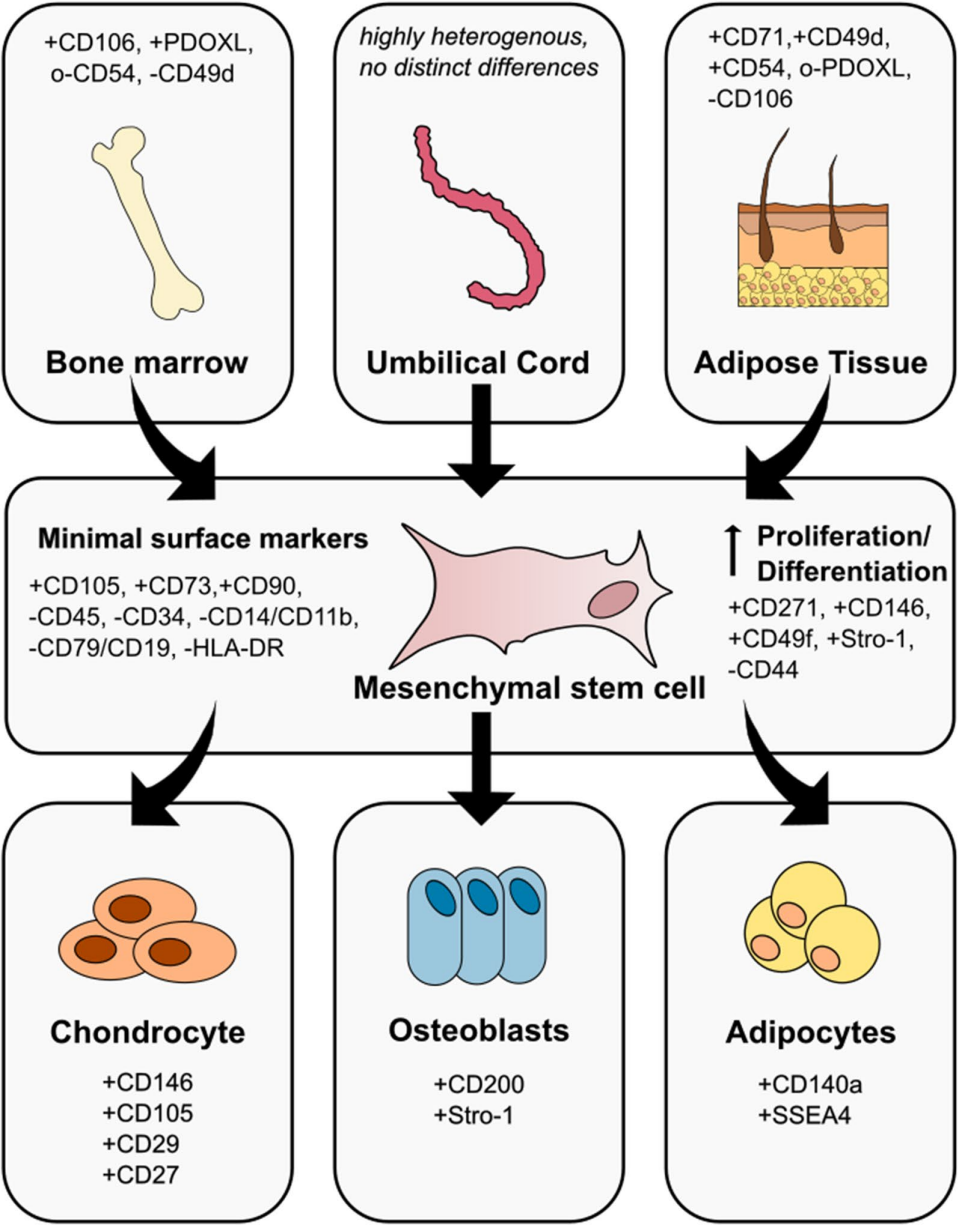
Surface markers of mesenchymal stem/stromal cells and the differences from various tissues and for different cell lineage commitments. + : high expression, o: moderate expression, -: low expression

MSCs isolated from bone marrow (BMMSCs) are most used in clinical trials, followed by cells from the umbilical cord (UCMSCs) or umbilical cord blood (UCBMSCs) and from adipose tissue (ADMSCs). BMMSCs have a higher chondrogenic potential than ADMSCs or UCBMSCs, but a lower proliferation rate. ADMSCs and UCBMSCs can be kept in culture for longer, the onset of senescence is later and they remain genetically and morphologically stable. [8] In addition, ADMSCs and UCBMSCs have higher immunomodulatory capabilities [9]. However, UCBMSCs are more heterogeneous than BMMSCs and ADMSCs due to a higher divergence between donors [5]. The cells can be distinguished by their surface markers as seen in Fig. 1. However, it is difficult to establish general valid criteria, as exempt subpopulations are always found.

Genetic manipulation could overcome the unsatisfactory performance by steering the cells to the desired phenotype and enhancing the desired traits or even introducing new factors and thus new cell functions. In general, co-transfection seems necessary for more complex treatments. However, transfection of MSCs is more challenging compared to transfecting cell lines, especially with multiple plasmids.. Nucleic acids must overcome several barriers, including the cell membrane, the endosomal escape and cytoplasmic transport, the escape from the vehicle, and the translocation into the nucleus. In addition, the transfection method may lead to cellular stress and may negatively affect cell metabolism and viability. As transfection efficiency is very high, viral vectors are most commonly used in clinical trials for gene therapy. However, the production of viral vectors on a commercial scale is relatively expensive and time-consuming, and there is still a small risk of triggering an immunogenic response or of mutagenesis, which needs to be monitored long-term. The use of non-viral methods circumvents these problems, but transfection efficiency and cell specificity are often insufficient.

## Heterogeneity and donor variability of MSCs

It is noticeable that only a few studies indicate transfection efficiencies for co-transfection as most studies focus on the application instead of the methodology (Tables 3 and 4). Furthermore, there are hardly any comparisons of transfection efficiencies between different donors or tissue sources. Especially comparisons of different studies and different laboratories are difficult due to the high plasticity of MSCs. Calcat-I-Cervera et al. [10] demonstrated, that MSCs derived from the same donor source and cultivated with the same protocol still showed differences in proliferation and differentiation when analyzed in different laboratories.

In general, transfection methods where the nuclear internalization of the nucleic acids is a limiting factor, such as lipofection and electroporation, should be more efficient on rapidly proliferating cells. However, some studies observed a better transfection efficiency of BMMSCS than of ADMSCs [11–13]. The donor variability therefore appears to have a more prominent influence on transfection efficiency than the tissue source and the question arises as to how many donors are needed to achieve a statistical representation.

For better overall performance, it therefore would be helpful to screen and sort MSCS populations for the presence of desired characteristics such as proliferation and differentiation potential, especially since co-transfection methods in particular are stressful for MSCs. Cells that have a high expression level of CD271 (low-affinity nerve growth factor receptor) and CD146 (melanoma cell adhesion molecule, MCAM, or cell surface glycoprotein, MUC18) show increased proliferation and a more pronounced trilineage differentiation, as do the markers CD49f (Integrin α-6) and Stro-1 (Stro-1 antigen) [14–18]. Furthermore, Kuci et al. [19] demonstrate that in selected and expanded CD271 + -cells, the expression levels for genes of the extracellular matrix and adhesion are increased, while they are decreased for genes of immunoregulatory processes. It is important to note that the markers were only observed in isolation.

However, since high cell numbers are required for clinical applicability, the feasibility depends on the frequency of CD-specific MSC subtypes within the mixed populations after isolation from a given cell niche (Table 1). Although there are some differences between the individual studies, it appears that BMMSCs are best enriched via the markers CD146 and CD49f and UCMSC via CD146 and Stro-1. ADMSCs only show a moderate expression level for all markers, so a combined enrichment would be best.

**Table 1:** Expression level of surface markers known for increased proliferation and differentiation potential from different MSC sources

Surface marker	Tissue	Donor	Passage	Expression	References
CD271	BMMSCs	n = 12, female, 31–40 y	n.i	6 ± 11%	[20]
		n = 8, 33 ± 20 y	3	3.7 ± 2.2%	[15]
		n.i	n.i	29.13 ± 8.18	[14]
	ADMSCs	n = 12, female, 27–35 y	n.i	5 ± 2	[20]
		n = 8, 55 ± 5 y	n.i	8.4 ± 4.6%	[15]
		n.i	n.i	89.20 ± 5.66%	[14]
	UCMSCs	n = 8	n.i	< 0.5%	[15]
CD146	BMMSCs	n = 12, female, 31–40 y	n.i	99 ± 0%	[20]
		n = 8, 36.5 + -9.8	n.i	50.14 ± 15.50%	[21]
		n.i	3	16.36%	[22]
	ADMSCs	n = 12, female, 27–35 y	n.i	38 ± 24%	[20]
		n = 2, 25–45 y	2	32.6%	[16]
	UCMSCs	n = 3	n.i	66%	[23]
		n.i	n.i	43.25%	[24]
CD49f	BMMSCs	n = 12, female, 31–40 y	n.i	85 ± 10%	[20]
		n = 3	1–2	11%	[17]
	ADMSCs	n = 12, female, 27–35 y	n.i	6 ± 3%	[20]
	UCBMSCs	n.i	n.i	13%	[25]
		n.i	3	15%	[26]
Stro-1	BMMSCs	n = 9, 8–14 y	n.i	~ 50%	[27]
	ADMSCs	n = 9, 8–14 y	n.i	~ 30%	[27]
	UCMSCs	n.i	n.i	44.08%	[24]

## Co-transfection

In contrast to transfection with one nucleic acid, transfection with several plasmids and/or large plasmids poses a major challenge, as not all plasmids are taken up with the same efficiency. Primary stem cells, such as MSCs, are particularly difficult to transfect. Plasmid DNA uptake has a greater effect on MSC viability than on cell lines [28]. Vesicle escape and cytoplasmatic diffusion are more difficult, probably due to larger and more stable vesicles and a more rigid network in the hydrogel-like cytoplasm of MSCs [29]. In clinical applications, transfection with viral vectors is the most used method. MSCs express many amphotropic receptors and are therefore susceptible to viral transfection [30]. However, viral vectors are limited by the size of their cargo, so multiple vectors need to be transfected. Theoretically, non-viral vectors do not have this limitation, but the transfection efficiency is still much lower. Furthermore, transfection efficiency is in general determined by reporter expression with a single plasmid of about 5 kb, instead of using multiple or larger plasmids [31].

## Viral transfection methods

Most vectors for clinical application are based on adenoviruses, followed by retroviruses, lentiviruses and adeno-associated viruses (AAVs). Relevant characteristics are summarized in Table 2.

**Table 2:** Overview of the most commonly used viral vectors in gene therapy

	Retrovirus (commonly based on γ-retroviruses)	Lentivirus (based on HIV1)	Adenovirus	Adeno-associated virus (AAV)
Type/Description	ssRNA of 7–12 kb	ssRNA of 7–12 kb	linear dsDNA of 36 kb	ssDNA of 8 kb
	Genome	Genome	Genome	Genome
	*gag* (structural proteins), *gol* (enzymes for replication & integration), *env* (envelope proteins), flanked by LTR	*gag, gol, env, tat (*transactivator of transcription), *rev* (nuclear export of unspliced or partially spliced transcripts) and other accessory genes flanked by LTR	Packaging signal, early (E1-4) genes, late (L1-5) genes, flanked by ITRs	*rep* (coding for replication and integration), *cap* (proteins of the icosahedral nucleocapsid), flanked by ITRs
Host	Dividing cells	Dividing and non-dividing cells	Dividing and non-dividing cells	Dividing and non-dividing cells
Integration	Chromosomal	Chromosomal, but: episomal with integrase-deficient vectors	Episomal	Episomal (only WT chromosomal)
Packaging Capacity	< 9 kb	< 9 kb	< 12 kb	< 5 KB
Immunresponse	Low	Low	High	Moderate
References	[32–35]	[32, 36, 37]	[38, 39]	[38, 40, 41]

**Retroviruses** have a diploid single-stranded RNA genome of 7–12 kb in length, which is transcribed into a DNA intermediate (provirus) via a reverse transcriptase and randomly integrated into the host cell genome. Commonly used retroviruses are based on gamma retroviruses (often abbreviated as retrovirus) or **lentiviruses**, which are somewhat more complex in structure and have additional regulatory and accessory genes. Klicken oder tippen Sie hier, um Text einzugeben.Therefore, lentiviruses can also infect non-dividing cells. Since human cells do not have receptors for the envelope glycoprotein, they must be pseudotyped with the vesicular stomatitis virus glycoprotein G (VSV-G). This allows the virus to infect virtually any mammalian cell. In addition, VSV-G pseudotyped retroviruses have a higher particle stability, which allows concentration by ultracentrifugation [35]. Nevertheless, the titer is lower than with other viruses, especially for the third generation, where the probability of recombination between the transfer and packaging plasmids to form a replicable virus is very low [36] In addition, integrase-deficient lentiviral vectors for transient gene expression have also been developed. The target cells are usually transfected in vitro and implanted afterwards. Single or co-transfection efficiencies of MSCs are generally about 80% [42]. However, Lin et al. [43] report that the transfection efficiency of BMMSCs was highly donor dependent, with efficiencies ranging from 39 to 89% at passage 1 with a multiplicity of infection (MOI) of 5. Integrating lentiviruses are the most used viral vectors for co-transfection.

**Adenoviruses** are widespread and over 50 different serotypes have been classified. Most vectors are based on adenovirus type 5 (Ad5). Adenoviruses transfect both dividing and non-dividing cells and do not integrate into the host genome (transient gene expression) [38]. For gene transfer, replication incompetent vectors are produced with a packaging capacity of up to 12 kb and a high titer of 1 × 10^13^ can be produced. In clinical applications, adenoviruses are mainly used for vaccination and oncolysis, as they induce a strong immune response, which can lead to inflammation and a shortened expression time [39]. Single transfection efficiencies of 80% are reported for BMMSCs [44, 45]. No transfection efficiency is reported for co-transfection, although they are used for this purpose (Table 3). However, it seems unlikely that the transfection efficiency will drop significantly.

**The adeno-associated virus** requires helper viruses (originally adenoviruses, hence the name), which provide the proteins for replication in the host cell. Integration of the wild type occurs specifically at the AAvS1 site on chromosome 19, but the DNA is usually present extrachromosomal in the replication-incompetent vectors [41]. The packaging capacity is limited to 4–5 kb [38].Yao et al. [46] report a single transfection efficiency of roughly 70% with serotype 2 and an MOI of 10,000 for BMMSCS 15 days after infection, which could be further increased with increasing MOI. In addition, the differences between donors had a significant impact on transfection efficiency as long as the MOI remained below 50,000. Donor variability was also demonstrated for ADMSCs with transfection efficiencies ranging from roughly 48% -72% 3 days after infection with an MOI of 10,000 [47]. For co-transfection, no potential clinically relevant cases were found. However, just as with the adenoviruses it seems unlikely that the transfection efficiency will drop significantly.

Overall, transfection efficiency is mostly dependent on the MOI, i.e. the number of infectious particles relative to the number of host cells. Donor variance only plays a role with low MOIs. Therefore, co-transfection regardless of tissue source is most efficient with viral vectors. Lentiviruses are the most suitable for the clinical applications mentioned here since they are less immunogenic than adenoviruses and have a higher packing capacity than AAVs. However, in some studies, transfection efficiency is enhanced by adding polybrene, a cationic polymer that neutralizes the charge repulsion between the virus particle and the host cell surface. There is some controversy about polybrene, as it could inhibit cell proliferation at low concentrations of 1 µg/ml and exposure time of 6 h [48, 49].

## Non-viral transfection methods

### Nanocarriers

Nanocarriers form complexes with nucleic acids by electrostatic interactions and are taken up by cells via endocytosis or direct membrane fusion. They are very reactive due to their large surface area in relation to their volume and can be manufactured in a wide range of organic and inorganic materials. Common organic materials for MSC transfection are lipids, polymers (PEI [50], PLGA [51]), polysaccharides (dextran, chitosan [52]), and peptides (RALA [50]), while silicium oxide [53] and iron oxide [54] are mostly used for inorganic materials. Currently, co-transfection is mainly carried out using lipids as nanoparticles in the literature, therefore only lipofection is described in more detail here.

Cationic lipids consist of a hydrophilic head group connected by a linker to a hydrophobic tail group. Each domain contributes to transfection efficiency and, in theory, can be specifically selected and modified depending on the application and cell type. However, since the efficiency is more than the sum of the individual domains, an optimal formulation and systematic comparison turns out to be very complex and still leaves much room for improvement [55]. Depending on the formulation of the lipids, liposomes, micelles, or densely packed lipid nanoparticles are formed.

For liposome formation, mainly synthetically produced, cationic lipids are used, whose hydrophilic head group consists monovalent of quaternary ammonium salts (e.g., DOTMA, DOTAP) or multivalent of primary and secondary amines (e.g., DOSPA, DOGS). The hydrophobic tail consists of saturated or unsaturated hydrocarbon chains [56]. However, the permanent positive charge of the head group can lead to interference with signaling pathways and enzymes, so many lipoplexes show dose-dependent toxicity [57].

Boura et al. [11] reported transfection efficiencies of 58 ± 7.1%, 54 ± 3.8% and 33 ± 4.7% for BMMSCs, UCMSCS and ADMSCs, respectively, however using only one single donor. Bakhshandeh et al. [58] achieved a transfection efficiency of 47% for UCBMSCs, while for BMMSCS only 3.67% of the cells were transfected. Cheung et al. [59] achieved transfection efficiencies of BMMSCS with 5 different donors of 24–36% by lipofection with TransIT-2020. For single transfection of MSCs Kozisek et al. [12] had transfection efficiencies around 30–45%, with different transfection efficiencies across two different donors per tissue source and overall higher transfection efficiencies of BMMSCS compared to ADMSCs. However, transfection with more than one plasmid reduces transfection efficiency by at least 10%. Therefore, lipofection is not yet suitable for co-transfection.

### Electroporation

During electroporation, the cells are exposed to a temporary electric field, which leads to a short-term depolarization of the membrane and thus to permeabilization through pore formation and other structural changes [60]. The critical voltage that must be reached depends on the cell type and therefore the cell size and membrane curvature, as well as the size and charge of the molecule to be transported. The efficiency depends on the pulse shape, the pulse length, the field strength, the number of pulses, the buffer, the temperature, and the number of cells [61]. Due to the large number of parameters, very variable transfection efficiencies are specified in the literature. Liew et al. [13] achieved a single transfection efficiency for BMMSCs with five different donors of 79% with low donor variance and for ADMSCs of 69%, with higher donor variability of around 15% difference between two donors. No–co transfection efficiencies are reported.

Two advanced procedures that promise greater transfection efficiency are Nucleofection and Microporation**. Nucleofection** was developed and patented by Lonza Cologne AG in 2001. Optimized electrical parameters and cell type-specific buffers allow the plasmid DNA to enter the cell nucleus directly. As a result, the transfection efficiency is independent of cell division and enables transfection even of non-dividing primary cells. For MSCs, a transfection efficiency of around 70% is reported 72 h after transfection with a GFP reporter plasmid for BMMSCS and ADMSCs [62, 63]. However, Haleem-Smith et al. [64] reported differences in transfection efficiencies from different donors of BMMSCs with a maximal difference of 20%. No transfection efficiencies are reported for the transfer of two plasmids, but the technology is successfully used for CRISPR/Cas9 applications.

For **microporation,** a pipette tip is used as the electroporation chamber and a capillary electrochamber instead of an electroporation cuvette. This avoids variations in temperature and pH value as well as the formation of metal ions. Comparing electroporation, nucleofection and microporation of UCBMSCs, Yeon Lim et al. [65] reported a single transfection efficiency of around 40%, 50% and 80% respectively. Microporation seems to be the most efficient non-viral method for co-transfection with an efficiency of 78% (Table 3). However, the method needs to be scaled up for clinical applicability.

Overall, non-viral methods are more dependent on donor variability and tissue source than viral methods and therefore have a lower potential for co-transfection. Probably because the transfection efficiency is dependent on many more parameters like plasmid size and amount, cell source, cell passage, density, proliferation rate and media components and therefore transfection efficiencies vary greatly in the literature. There is ongoing research to improve the non-viral transfection efficiency. Besides the improvement of the transfection reagent itself, is to prime the MSCs beforehand with hypoxia or with glucocorticoids like dexamethasone [66] or to stimulate the cells with interferon-gamma for an enhanced immunomodulatory ability. Another way is to reduce the plasmid size by deleting the bacterial backbone, which results in minicircles [67]. For a detailed discussion the reader is referred to [68, 69].

## Preclinical applications of co-transfected MSCs

The preclinical applications of co-transfected MSCs are summarized for viral methods (Table 3) and non-viral methods (Table 4).

**Table 3:** Viral co-transfection in mesenchymal stem/stromal cells and their applications

Method	MSC type and passage	MOI	Genes	Transfection efficiency	Readout	Application	References
Adenovirus	C57BL/6 J mice, bone marrow, P3, male	600	PDX-1 NeuroD1	Single: 80%	Insulin expression with at least a 110-fold increase over control	Differentiation into insulin-producing cells	Qing-Song et al. [44]
			MafA				
Adenovirus	Wistar inbred rats, bone marrow, P4, male	100	VEGF	Single: 92%	VEGF and SDF-1 expression with a sixfold increase over control	Improved cardiac function after myocardial infarction	Tang et al. [45]
			SDF-1				
Lentivirus	Sprague Dawley rats, bone marrow, P3, male	10	BDNF	n.i., only fluorescence images	BDNF/VEGF mRNA levels with at least an 80/160-fold increase over control	Enhanced neuroprotection after cardiac arrest-induced global cerebral ischemia injury	Zhou et al. [70]
			VEGF				
Lentivirus	Sprague Dawley rats, adipose tissue, P2, male	100	VEGF	n.i., only fluorescence images	VEGF/GDNF expression with at least an 8.5/4.5-fold increase over control	Treatment of neurogenic erectile dysfunction	Yang et al. [71]
			GDNF				
Lentivirus	Sprague Dawley rats, bone marrow, P3, male	n.i	BDNF	n.i., only fluorescence images	BDNF/GDNF mRNA levels with a twofold increase over control	Repair of peripheral nerve injury	Zhang et al. [72]
			GDNF				
Lentivirus	Human, adipose tissue, P3-6	20	Oct4	n.i	Relative Oct4/Sox2 levels with 1.7/13.4-fold increase	Anti-inflammatory effects	Li et al. [73]
			Sox2				
Lentivirus (screened with 2 µg/ml puromycin and 15 µg/ml neomycin)	Sprague Dawley rats, adipose tissue, P3, male	40	VEGF	n. i., only fluorescence images	VEGF and Smad7 expression levels with at least a threefold increase over control	Treatment of neurogenic erectile dysfunction	He et al. [74]
			Smad7				
Lentivirus (screened with 2 µg/ml puromycin)	Sprague Dawley rats, bone marrow	200	VEGF	Single: 70%	VEGF/Bcl-2 expression with a 1.8/threefold increase over control	Enhancement of survival and the paracrine effect	Ni et al. [75]
			Bcl-2				
Lentivirus (supernatant + 2 µg/ml polybrene)	Sprague Dawley rats, bone marrow, P3-5, male and female	n.i	NGF	n.i	NGF/bFGF expression levels around 9/11-fold increase over control	Enhancement of neural differentiation	Hu et a., [76]
			bFGF				
Lentivirus (supernatant + 40 µl HitransG P	Sprague Dawley rats, bone marrow, P2, male	successive 10 + 15	CXCR4	n.i., only fluorescence images	CXCR4/IL-35 expression around a 4.5/tenfold increase over control	Enhancement of migration and immunoregulatory capacity	Tan et al. [69]
			IL-35				
Lentivirus (supernatant + 5 µg/ml polybrene)	Wistar rats, bone marrow, P2	80	BMP2	Single: 90%	BMP2 and Ang-1 concentration with at least a 1.8 ng/ml	Osteogenic differentiation for bone regeneration	Liu et al. [49]
			Ang-1				
Lentivirus (supernatant + 5 µg/ml polybrene, screened with 1 µg/ml Puromycin)	C57BL/6 J mice, bone marrow, P2-3, female	30	VEGF	Single: 80–90%	VEGF and BMP2 mRNA expression with at least a 22-fold increase over control	Bone regeneration of calvarial defects	Guo et al. [77]
			BMP2				
Lentivirus (supernatant + 5–10 µg/ml polybrene, screened with 300 µg/ml G418 and 0.15 µg/ml puromycin)	Human, Wharton jelly,	n.i	TH	n.i., only fluorescence images	Dopamine expression of 0.5179 ± 0.0522 pg	Dopamine synthesis for the treatment of Parkinson’s disease	Li et al. [78]
			AADC				
			GCH1				
Lentivirus (supernatant + 6 µg/ml polybrene)	ALB/c and C57BL/6 mice, bone marrow, P4-8, male	100	IL-4	84.2% of BALB/c MSCs and 79.4% of C57BL/6 MSCs	IL-4 and PDGF-BB expression with 2500 pg/ml and 75 pg/ml	Enhancement of bone regeneration	Zhang et al. [42]
			PDGF-BB				
Lentivirus	Sprague Dawley rats, bone marrow	n.i	Optogenetic system Ilhx8 BMP2	n.i	BMP2 mRNA expression with at least a 17-fold increase and LHX8 expression with at least threefold decrease over control	Enhancement of bone regeneration	Wang et al. [79]
Adenovirus	Human, umbilical cord	n.i	Optogenetic system CIBN-EGFP-CD9 NOS-mCherry-CRY2	n.i	No quantitative protein expression. Enriched exosomes	Enriched exosomes for improved wound healing	Zhao et al. [80]

**Table 4:** Non-viral co-transfection in mesenchymal stem/stromal cells and their applications

Vector	MSC type and passage	Genes	Method	Transfection efficiency	Readout	Application	References
Chitosan nanoparticles	Humane, adipose tissue, female	BMP2	5915 bp	19.03% ± 1.74%	Protein expression with a 1.47 and 1.49-fold increase over control	Increase of osteogenesis	Hu et al. [52]
		FGF	5181 bp				
Lipofection: Lipofectamine 2000	Goat, bone marrow, P4, male	STRA8	5870 bp	n.i, only fluorescent images	mRNA expression with ~ 10,000-fold, 450-fold and 7000-fold increase over control	Differentiation into putative male germ cells	Zhang et al. [81]
		BOULE	5590 bp				
		DAZL	n.i				
Microporation (Invitrogen)	Human, bone marrow, P3	SOX-5	0.17 µg each	78.3 ± 6.5%	mRNA expression with a 115-fold, 20-fold and 100-fold increase over control	Enhancement of chondrogenesis	Kim et al. [82]
		SOX-6					
		SOX-9					
Electroporation	Umbilical cord	CRISPR/cas9	29.2 µg	n.i	Protein expression with a twofold increase over control	Upregulation of endogenous BMP4 for osteogenic differentiation	Choi et al. [83]
		dAsCpf1-VPR sgBMP4	29.3 µg				
Lipofection: Lipofectamine 2000	C56BL/6 mice, bone marrow, male	CRISPR/Cas9	1 nM	n.i	Protein expression with at least a twofold increase over control	Upregulation of Interleukin-10 for suppressing inflammatory response in myocardium	Meng et al. [66]
		dCas9-VP64-MS2/gIL10	1 nM				
		With MS2-p65-HSF1					
Nucleofection	C57BL/6N mice, umbilical cord, male	CRISPR/Cas9	15 µg Cas9 protein	n.i	Protein expression with a 45.9-fold increase over control	Enhancement of neuronal protection and survival	Lee et al. [84]
		pZDonor-AAVS1 Puromycin	20 µg gRNA				
		RNP	1 µg donor DNA				
		gsRAGE					
Nucleofection (selection with 1 µg/ml puromycin)	Human, umbilical cord,	CRISPR/cas9	15 µg Cas9 protein	n.i	Protein expression with a 5.86-fold increase over control	Promoted cell survival and enhanced regeneration of brain tissue for Rett syndrome disease	Kim et al. [85]
		pZDonor-AAVS1 Puromycin	20 µg gRNA				
		RNP	1 µg donor DNA				
		gsBDNF					

### Maintenance of stemness and differentiation potential

For clinical applications high cell numbers are required. However, during in vitro expansion MSCs lose their stemness properties and differentiation potential progressively [86]. Co-expression of pluripotent specific factors can attenuate the progress. Co-expression of Oct4 and Sox2 promotes cell proliferation and increases the differentiation potential [73, 87]. Moreover, anti-inflammatory effects were enhanced compared to non-transfected MSCs. Expression of the anti-inflammatory cytokine IL-10 was up-regulated, while TNF-α was downregulated.

Another aspect to overcome is the low survival rate of transplanted cells. Overexpression of VEGF and Bcl-2 reduced apoptosis, decreased autophagy and enhanced the paracrine effect [75].

### Neuronal protection and regeneration

MSCs provide neurotrophic factors and cytokines to promote the repair and regeneration of impaired neurons as well as decreasing apoptosis and regulating inflammation. Overexpression of neurotrophic factors like BDNF and VEGF enhance the neuroprotective efficacy. Zhou et al. [70] co-transfected BDNF and VEGF in BMMSCs and injected them in a cardiac arrest mouse model. Overexpression leads to enhanced protection of neurons and enhanced angiogenesis associated with neurofunctional improvement after seven days compared to naive BMSCs.

A synergistic effect of BDNF and GDNF on nerve repair efficiency of the damaged sciatic nerve of SD rats was demonstrated by Zhang et al. [72]. They suggest that, combinations of neurotrophic factors are more effective than single neurotrophic factors. Different mechanisms of action are triggered, although doses are generally lower compared to single neurotrophic expression.

Instead of transfecting recombinant genes, Hsu et al. [88] use CRISPR/Cas9 technology to activate and enhance endogenous BDNF, GDNF, and NGF levels in ADMSCs for the repair of sciatic nerve injury.

Co-expression of BDNF with BCLXL improved resistance to apoptosis-inducing toxicants, thereby increasing survival rates after transplantation [89]. Other studies focus on differentiating MSCs into neuron-like cells before transplantation to enhance their paracrine effects [90, 91]. Co-transfection of BMMSCs with NGF and bFGF (also known as FGF2) promotes neural differentiation indicated by the expression of neuronal markers like nestin, NSE, GFAP and ß-tubulin III [76].

### Cavernosum nerves

Radical prostatectomy to remove cancerous tissue can lead to cavernous nerve damage which is the cause of erectile dysfunction due to fibrosis. Injection of MSCs promotes repair of the damaged cavernosum to a certain extent. However, genetic manipulation of MSCs to overexpress VEGF and Smad7 has a significantly stronger effect on improving erectile function than untransfected or single-transfected MSCs [74]. Similar results could be achieved with VEGF and GDNF overexpression [71].

### Bone and cartilage repair

An already successful application of MSCs is the site-directed transplantation for bone and cartilage repair of bony defects caused by trauma, infection, or cancer. MSCs are often transfected with bone morphogenetic protein 2 (BMP-2) to differentiate MSCs into chondro -or osteocytes in vitro beforehand [92]. However, promoting vascularization enhances bone regeneration. Hu et al. [52] demonstrate that co-transfection of BMP2 and FGF2 results in a synergistic effect on osteogenesis. Expression of the osteogenesis markers BSP and OCN were at least 1.6-folds higher compared with single-gene transfection. Co-transfection increases angiogenesis and calcium deposition [48]. Similar results were shown with Angiopoietin-1 instead of FGF2 [49]. Another strategy is the co-overexpression of interleukin-4 (IL-4) and platelet-derived growth factor (PDGF)-BB [42]. While IL-4 decreases inflammation, it can also inhibit the osteogenesis of MSCs. Therefore, co-expression with PDGF-BB reduces the inhibitory effect leading to increased cell viability, proliferation and osteogenesis in the acute inflammatory phase.

### Treatment of diabetes

Type 1 diabetes is caused by T-cell mediated destruction of pancreatic beta-cells and the resulting insulin-deficiency. Cell replacement is limited by a lack of pancreas donors, therefore genetic manipulation of MSCs into insulin-producing cells seems to be a promising approach. Qing-Song et al. [44] transiently transfected murine BMMSCS by adenoviral transfection with a combination of PDX-1, NeuroD1, and MafA and could show that the amount of produced insulin is threefold higher when all genes are transfected together compared to one or two transfected genes. Blood glucose levels after transplantation of the transfected cells into streptozotocin-induced diabetic mice showed nearly the same levels as the control with transplanted beta-cells. However, the effect is reduced after 14 days because of transient transfection.

### Mycoardical infarction

Cardiovascular diseases, including myocardial infarction (MI) are the leading cause of death worldwide. Gene therapy seems promising for promoting myocardial regeneration and reducing fibrosis after acute myocardial infarction. Meng et al. [66] use CRISPR/Cas9 to overexpress Interleukin 10 in BMMSCs and transplant these cells afterwards in diabetic MI mice. They could demonstrate that IL-10 overexpression suppressed inflammatory cell infiltration and reduced inflammatory cytokine expression at least threefold, thereby improving cardiac performance. To improve the homing to the inflamed area, Hervas-Salcedo et al. [67] co-transfect IL-10 mRNA together with CXCR4 mRNA. CXCR4, a chemokine receptor binding to SDF-1, promotes migration to the injury sites [68]. After injection in a mouse model with an induced inflammation of the right pad, twice as many MSCs could be observed after 24 h compared to untransfected MSCs. Similar results could be shown in vitro by co-expressing CXCR4 with IL-35 [69]. Tang et al. [45] co-transfected VEGF and SDF-1 in BMMSCs. Transplanted cells show not only a synergistically increased expression and an improved survival rate in comparison to untransfected and single transfected cells, but also a reduction in infarct size and fibrosis.

### Optogenetics

Optogenetic systems originated in the manipulation of light-activated ion channels, but have also progressed to a spatiotemporal control of gene expression. The system is based on a photoreceptor that can only interact with a specific binding partner when activated by light of a specific wavelength. Each of these proteins are associated with a transcription factor, which combine to form a functional unit to activate the promoter of a specific target sequence [93]. Potentially, this allows for a simulation of naturally occurring changes in gene expression, which is demonstrated by Wang et al. [79]. They use the optogenetic FKF1/GI system to control the expression of BMP2 and Lhx8 in the early and late stages. The expression of Lhx8 promotes BMMSC proliferation in the early stages, while upon light illumination the expression is inhibited and the expression of BMP2 for cell differentiation is started. Zhao et al. [80] use the EXPLOR system to enrich UCMSC-derived exosomes with eNOS for improved diabetic wound repair. In a high-glucose environment, the enriched exosomes promote survival and migration of HUVECs and Fibroblast.

## Evaluation/risks of genetically manipulated MSCs in clinical therapies

For unmodified cells, Thompson et al. [93] conclude in a meta-analysis of 55 randomized controlled trials that there is no correlation between MSC therapy and infection, malignancy, development of thrombotic or thrombo-embolic events, and non-fever acute infusion toxicity. On the contrary, after MSC treatment the probability of dying is lower compared to the control group. Only seven patients suffered severe adverse effects related to MSCs treatment, e.g., acute in-stent thrombosis and acute coronary artery occlusion due to MSC diameter. However, there is a significantly higher risk of fever after MSC treatment compared to the control group. In addition, only a few studies provide information on the viability and analysis of surface markers in terms of potency and functionality.

Precise causal research is proving difficult due to the many variable parameters affecting the efficacy of MSC transplants like MSC origin, donor (autologous or allogeneic, matched or unmatched), administration route, dosing, different culture media with partly xenogeneic compounds, using freshly isolated cells or cryopreserved cells and expansion time. It is therefore not surprising that a lack of standardized methods leads to diverse results in clinical trials. The culture period to obtain sufficient MSC numbers, especially BMMSCs, may invoke genetic changes with a change in the polyclonal composition [94] and may lead to increased senescence with an impairment of their functionality and/or increased production of pro-inflammatory cytokines [95]. There is also a discussion about the influence of cryopreservation on efficacy. While marker expression and differentiation potential remain unchanged, some studies report that thawed BMMSCs exhibit lower inhibition of T cells [96]. However, other studies could not demonstrate this effect. Therefore, limited efficacy could not only be because of low retention time in the bodies but also because of insufficient cultivation and functionality assays beforehand. Indeed, senescence is often overlooked and should be evaluated extensively. For example Bertolo et al. [97] developed a score set to quantify the senescent state of BMMSCs correlated to the differentiating capacity based on colony-forming unit (CFU) assay, population doubling time (PDT), senescence-associated β-galactosidase (SA-β-Gal) activity, cell size, telomere length and gene expression of MSCs cultured in vitro over 11 passages. Such a set could also be extended for other desirable functions like a T cell proliferation test for immunosuppression activity and would likely be tissue source-specific.

On top of that, for genetically engineered MSCs, it must be proven that the overexpression of transgenes does not result in unwanted side effects. In addition, the transfection method and the plasmid backbone could also impact gene expression undesirably.

Comparing the four non-viral and transient delivery methods polyethyleneimine (PEI), cationic liposome, calcium phosphatase nanoparticles, and microinjection, Guan et al. [98] show that the methods affect the differentiation potential of MSCs to varying degrees in vitro. While transfection with calcium phosphate promotes osteogenesis and reduces adipogenesis, transfection with PEI greatly reduces osteogenesis and promotes adipogenesis. Microinjection and lipofection show no influence on osteogenic or adipogenic potential. Furthermore, Gonzales-Fernandez et al. [50] demonstrate that the choice of transfection method has a greater effect on differentiating capability than the expression level of osteogenesis or chondrogenesis promoting transgenes BMP-2 and TGF-β3. This is mostly due to the morphological changes during transfection, which regulate lineage commitment in addition to cytoskeletal tension and focal adhesion [99].

A lot of studies demonstrate no negative effects on the differentiating capacity of MSCs after viral transfection, although a round morphology is often observed after transfection [44, 100–102]. However, only a few studies investigate the gene expression profiles after transfection. Wang et al. [103] have found a lot of genes that are differently expressed in BMMSCs after lentiviral transfection (second generation) with a fused reporter gene containing functional domains from firefly luciferase, monomeric red fluorescent protein and a truncated mutant herpes simplex virus 1 thymidine kinase compared to non-transfected cells or transfected cells with an empty vector, e.g. genes associated with stem cell development, immune response, protein expression and metabolism. While transfected cells show no differences in the common marker expression for cell differentiation compared to transfected cells with an empty vector or non-transfected cells, some genes regulating lipid metabolism or ossification and cartilage formation were differently expressed. This could be the reason for an enhanced adipogenic and osteogenic differentiation potential after transfection with the fused reporter. In addition, cells with high reporter expression have a decreased proliferation rate, which could also be shown at the gene level. Interestingly, cells that were only transfected with the backbone share only a few genes that were similarly expressed to the transfected cells with the reporter gene. It may be reasonable to assume that changes in gene expression are dependent on the introduced transgenes and not on the viral vector.

Overall, MSCs need to be evaluated before and after transfection to see whether differences can have a negative effect.

## Conclusion

Pre-clinical studies show the relevancy of genetically engineered MSCs in vitro to enhance cellular functions and improve future clinical outcomes. Expression of multiple genes not only promotes the differentiating capacity, anti-inflammatory properties or angiogenesis, but also promotes survival rate after transplantation and homing to the inflamed area. Furthermore, there is often a synergistic effect on gene expression, and different mechanisms can be triggered. The transfection method of choice is still by viral vectors. With the development of 3rd generation vectors and self-inactivating vectors the risk of mutagenesis is very low and mostly dependent on the transgene. Microporation and nucleofection are promising non-viral methods, however a high-throughput needs to be developed. Transfection alone and the introduction of a recombinant gene exerts stress on the cell and influences gene expression levels for many pathways. However, no relevant negative effects have been observed to date. Still, to exclude insertional mutagenesis after transfection, cancer-related mutations and chromosomal aberrations should be analyzed beforehand by gene expression analysis. Furthermore, long-term monitoring of patients receiving genetically engineered cells is very important.

To increase the success of a clinical trial and make it more comparable to each other, it is also important to reduce the variable parameters. Like many researchers said before, culture conditions and transfection methods should be standardized. On top of that, MSCs should be carefully screened beforehand, and used subpopulations need to be characterized.

## Data Availability

Not applicable.

## Abbreviations

AD: Adipose tissue
ANG-1: Angiopoietin 1
Bcl-2: B-cell lymphoma 2
BCLXL: B-cell lymphoma-extra large
BDNF: Brain-derived growth factor
bFGF/FGF2: Basic fibroblast growth factor/ fibroblast growth factor 2
BM: Bone marrow
BMP-2: Bone morphogenetic protein 2
BSP: Bone sialoprotein
Cas9: CRISPR-associated protein 9
CRISPR: Clustered regularly interspaced short palindromic repeats
CXCR4: C-X-C chemokine receptor type 4
DOPE: 1,2-Dioleoyl-sn-glycerol-3-phosphoethanolamine
DOSPA: 2,3-Dioleoyloxy-N-[2(sperminecarboxiamido)ethyl[-N,N-dimethyl-1-propaniminium trifluoroacetate
eNOS: Endothelial nitric oxide synthase
GDNF: Glial cell line-derived neurotrophic factor
GFAP: Glial fibrillary acidic protein
HDR: Homology directed repair
IL: Interleukin
Lhx8: LIM Homebox 8
MSCs: Mesenchymal stem/stromal cells
NeuroD1: Neurogenic differentiation 1
NGF: Nerve growth factor
NHEJ: Non-homologous end joining
NSE: Neuron specific enolase
OCN: Osteocalcin
Oct4: Octamer binding transcription factor 4
PDGF: Platelet-derived growth factor
PDX-1: Pancreatic and duodenal homeobox
PEI: Polyethylenimine
PLGA: Poly (lactic-co-glycolic acid)
RALA: Ras-related protein Ral-A
SDF-1: Stromal cell-derived factor 1
Sox2/SRY: Sex determining region Y-box 2
TNF-α: Tumor necrosis factor-alpha
UC: Umbilical cord
UCB: Umbilical cord blood
VEGF: Vascular endothelial growth factor
